# Zerumbone acts as a radiosensitizer in head and neck squamous cell carcinoma

**DOI:** 10.1007/s10637-021-01190-7

**Published:** 2021-10-06

**Authors:** Julia Schnoell, Isabella Stanisz, Bernhard J. Jank, Victoria Stanek, Rainer Schmid, Markus Brunner, Gregor Heiduschka, Ulana Kotowski

**Affiliations:** 1 Department of Otorhinolaryngology, Head and Neck Surgery, Medical University Vienna, Vienna, Austria; 2 Department of Radiotherapy, Medical University of Vienna, Vienna, Austria

**Keywords:** Zerumbone, Natural products, Head and neck squamous cell carcinoma, Cytotoxic, Radiation

## Abstract

*Introduction.* Zerumbone is a phytochemical compound of the ginger plant *Zingiber zerumbet* with cytotoxic effects in various cancer cell lines. To date, zerumbone has shown an antiproliferative effect in oral squamous cell carcinoma cells lines. However, the effect of combination with radiation or cisplatin in head and neck squamous cell carcinoma (HNSCC) is unclear. The aim of this study was to investigate the effect of zerumbone alone, and in combination with irradiation and cisplatin on HNSCC cell lines. *Methods.* The three HNSCC cell lines SCC25, Cal27 and FaDu were treated with zerumbone, radiation and/or cisplatin. Cell viability and clonogenic assays were performed. The interaction between zerumbone and radiation or cisplatin was evaluated using the combination index. Apoptosis was measured by flow cytometry and cell migration was assessed using a wound healing assay. *Results.* Treatment with zerumbone resulted in a dose dependent induction of cytotoxicity and apoptosis in all three cell lines. The combination with cisplatin revealed a synergistic to additive effect in Cal27. The clonogenic assay showed a significant radiosensitizing effect in all three cell lines. The wound healing assay showed a reduction of cell migration in Cal27. *Conclusion.* The natural compound zerumbone shows a cytotoxic and proapoptotic effect on HNSCC cell lines. Furthermore, zerumbone enhances the radiation effect in all three cell lines and thus may be a suitable candidate for combination therapy in HNSCC.

## Introduction

Head and neck squamous cell carcinoma (HNSCC) is the sixth most common cancer worldwide with an incidence of approximately 900.000 new cases per year [1]. The average 5-year survival rate of HNSCC is 66%, but strongly depends on the tumor stage and location [1–3]. The majority of patients are diagnosed with a locally advanced stage which requires a multimodal therapy including surgery, irradiation and/or chemotherapy. However, treatment intensification comes at the cost of adverse effects and is therefore limited [2–4]. Hence, the identification of suitable new substances is required which show antiproliferative effects and ideally help to improve radio- and chemotherapy.

Natural sources play a great role in the discovery of many anticancer drugs. For instance, taxanes and vinca alkaloids were initially isolated from the madagaskar periwinkle and the Pacific yew tree bark, respectively [5]. The advantage of natural products is that they often interact with multiple targets. Thus, they are more promising to show an effect in the dysregulated tumor cell state with alterations in hundreds of genes. Additionally, they are generally safer to use, less expensive and more accessible [6].

Zerumbone is a natural compound of the ginger plant *Zingiber zerumbet* Smith. In Asian traditional medicine the plant is used to treat a wide variety of diseases and symptoms. The anticancer properties of zerumbone have been reported in several studies in vitro and in vivo in various cancers [7–9]. In contrast to the cytotoxic effect on cancer cells, zerumbone shows only minimal to no effect on normal cells [9–11]. Other properties of zerumbone are anti-inflammatory, antioxidant, antiatheroslerotic, antinociceptive, antimicrobial and hepatoprotective activities [8]. Reported mechanisms leading to the antiproliferative effects of zerumbone include the modulation of the Nf-κB pathway, the mitochondrial pathway of apoptosis, upregulation of redox potential, inhibition of CXC chemokine receptor-4 expression, PI3K-mTOR and TRAIL pathway [7–9].

The aim of this study was to investigate the effect of zerumbone in the HNSCC cell lines SCC25, Cal27 and FaDu especially in combination with (the standard therapeutic options in HNSCC treatment) cisplatin and irradiation.

## Materials and methods

### Drugs

Zerumbone was obtained from Sigma Aldrich (St. Louis, MO, USA). It was dissolved in dimethyl sulfoxide (DMSO; Sigma Aldrich, St. Louis, MO, USA) as a 100 mmol/l stock solution and stored at -20 °C. Further dilution was done with RPMI medium immediately before treatment. Cisplatin (CIS) was taken from ready-to-use infusions.

### Cell culture

The HNSCC cell line FaDu was obtained from the American Type Culture Collection (Manassas, VA, USA). Cal27 and SCC25 were obtained from the German Collection of Microorganisms and Cell Cultures (Braunschweig, Germany). The cell line FaDu derived from a human pharyngeal squamous cell carcinoma whereas SCC25 and Cal27 are from human squamous cell carcinomas of the tongue. The cell lines were kept in RPMI medium (Gibco BRL, Gaithersburg, MD, USA) supplemented with 1% penicillin–streptomycin (Gibco BRL, Gaithersburg, MD, USA) and 5% fetal bovine serum (Gibco BRL, Gaithersburg, MD, USA) in a humidified atmosphere with 5% CO_2_.

### Cytotoxicity assay

A cytotoxicity assay was performed to assess the cytotoxic effect of zerumbone. 3 × 10^3^ cells were seeded on 96-well plates in triplicates and incubated for 24 h. Subsequently, they were treated with 0—96 µM zerumbone. For the combination experiments, cells were treated with cisplatin 0—32 µM or a combination of zerumbone and cisplatin (ratio 3:1) as well as with irradiation 0—8 Gy alone or in combination with zerumbone. 0,1% DMSO was used as vehicle control. After 72 h of incubation the percentage of living cells was assessed using the Cell Counting Kit-8 assay (CCK-8, Dojindo Molecular Technologies Inc., Rockville, MD, USA) according to the manufacturer’s protocol.

### Flow cytometry analysis

To assess the induction of apoptosis, flow cytometry analysis was performed. Briefly, 10^5^ cells were seeded on 6-well plates and incubated for 24 h. Then, cells were treated with 0, 4, 8 and 16 µM zerumbone. After 24 h and 48 h, apoptosis and necrosis were measured using the Annexin-V Apoptosis Detection Kit (Bender MedSystems, Vienna, Austria). Apoptosis was defined as Annexin + /propidium iodide − (Ann + /PI-) and necrosis/late apoptosis was defined as Ann − /PI + and Ann + /PI + .

### Wound healing assay

Cells were seeded on 6-well plates and after reaching a confluence of approximately 80%, a scratch was made using a 1 ml pipette tip. After washing twice with warm PBS, cells were treated with 0, 5, 10 and 20 µM zerumbone. Two regions per scratch were marked and photographed subsequently after the scratch and again after 24 h of incubation using CellSens Software (version 1.8.1, Olympus Corporation, Tokyo, Japan). The wound healing area was measured using ImageJ macro “MRI Wound Healing Tool” [12].

### Irradiation

Irradiation was carried out using a 150 kV x-ray machine (Gulmay D3300, Gulmay Medical Ltd., Byfleet, UK) at a dose rate of 2 Gy/min at room temperature. The focus-object distance measured 52 cm and the field size was 20 × 20 cm. Thermoluminescence dosimeters were used for dosimetry assessment.

### Clonogenic assay

The clonogenic assay was performed according to the protocol by Franken et al. [13]. Six-well plates were seeded with 3 to 15 × 10^2^ cells and incubated for 24 h. Next, cells were treated with 0, 2.5 or 5 µM zerumbone and/or irradiated with 0, 2, 4, 6 or 8 Gy and incubated for 72 h. Afterwards, the medium was replaced with drug-free medium. 10 days later, cells were washed with phosphate-buffered saline, fixed with ethanol 96% and stained with crystal violet. Colonies containing more than 50 cells were viewed as survivors and counted.

### Statistical analysis

All experiments were carried out at least three independent times. The statistical analysis for cytotoxicity assays and flow cytometry were performed using GraphPad 5.0 software by Prism® (GraphPad Software Inc., San Diego, CA, USA). The interaction of zerumbone and cisplatin and short term combination with irradiation were analyzed using the Combination Index according to the Chou-Talalay method using CompuSyn software (ComboSyn Inc.) [14]. The clonogenic assay was analyzed using a linear-quadratic model as described by Franken et al. [13]. Flow cytometry and wound healing assay were analyzed with GraphPad 5.0 software by Prism® using one-way analysis of variance (ANOVA) and Tukey's multiple comparisons test. A p-value below 0.05 was considered as statistically significant.

## Results

### Zerumbone leads to inhibition of cell viability

**Fig. 1:**
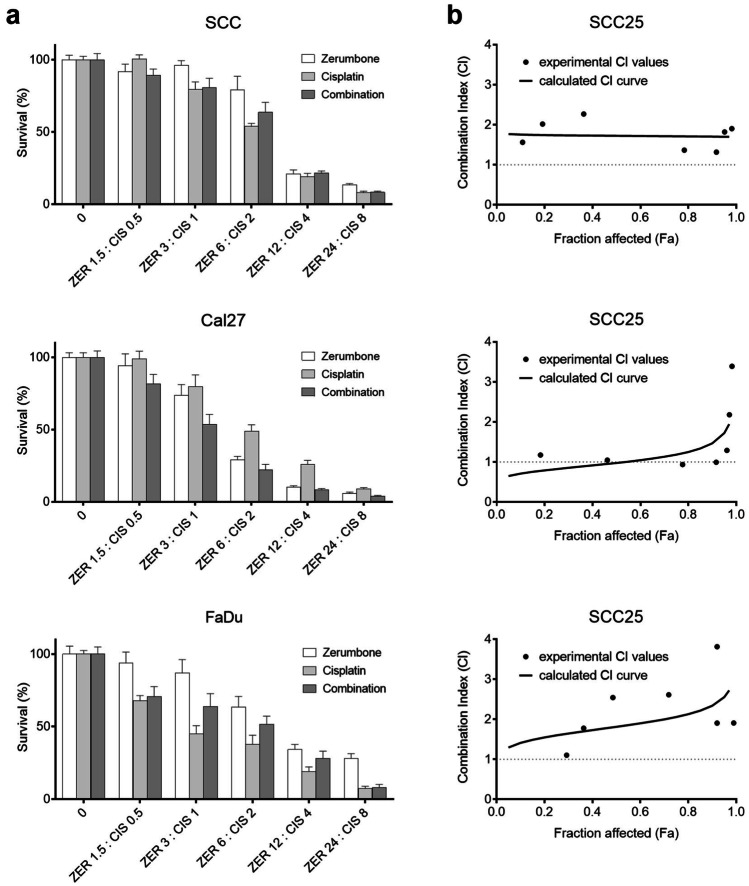
Combination of zerumbone and cisplatin. (**a**) Cell viability after treatment with zerumbone (ZER), cisplatin (CIS) or the combination of both for 72 h. Treatment concentration is described on the x-axis in µM. (**b**) Combination Index: CI < 1 indicates synergy, CI > 1 antagonism and CI = 1 represents an additive effect. Error bars represent the standard error of the mean

The three HNSCC cell lines SCC25, Cal27 and FaDu were treated with zerumbone (0–96 µM) for 72 h and cell viability was measured using CCK-8. Treatment with zerumbone showed a dose dependent inhibition of cell viability in all three cell lines (Fig. 1). Cal27 cells were most sensitive to zerumbone treatment with an IC_50_ at 4.42 µM, followed by FaDu cells with an IC_50_ at 8.60 µM. SCC25 was the least sensitive cell line with an IC_50_ at 9.22 µM.

### Treatment with zerumbone induces apoptosis

**Fig. 2:**
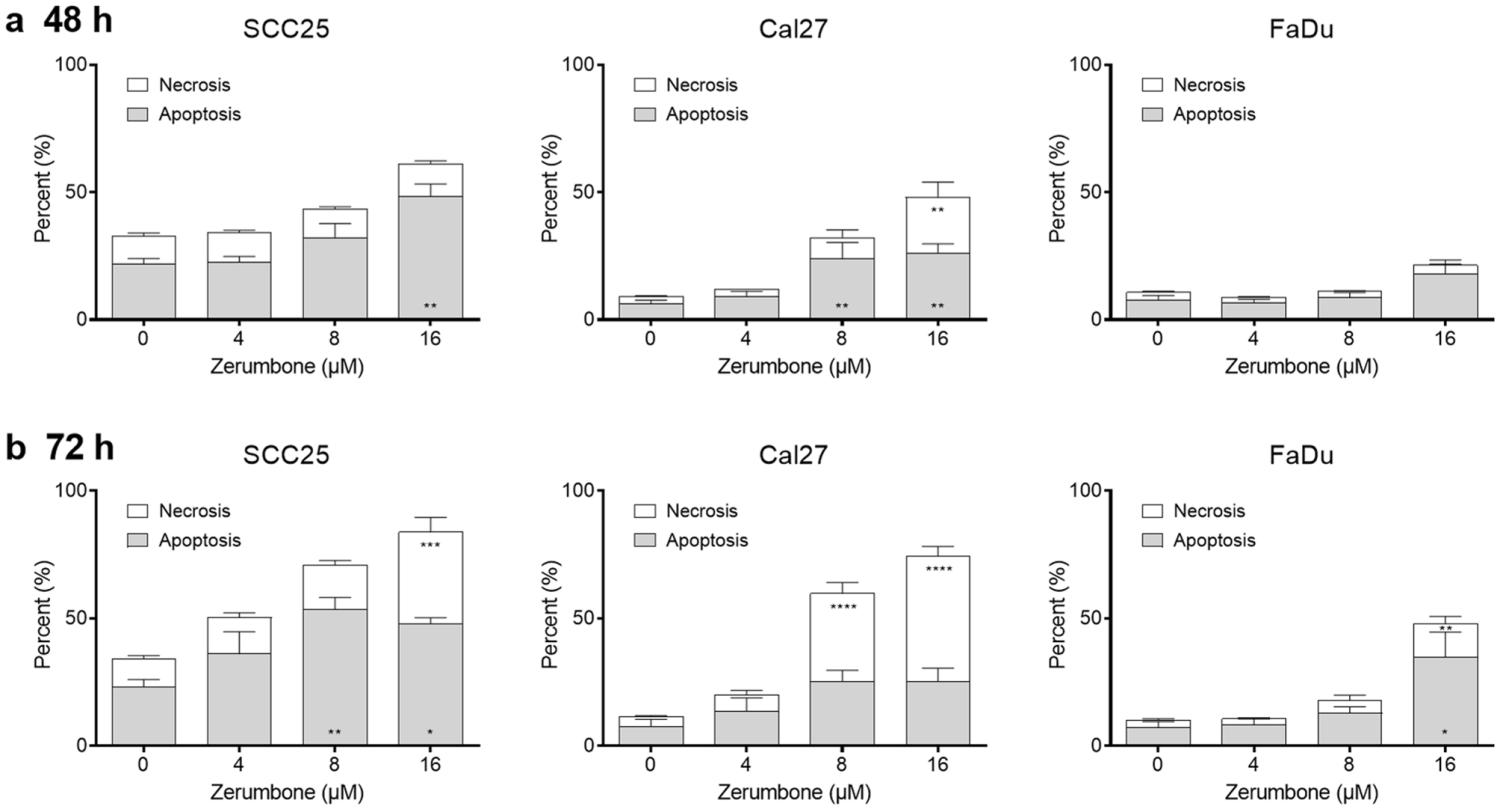
Flow cytometry analysis of apoptosis and necrosis after (**a**) 48 h and (**b**) 72 h of incubation with zerumbone. Percentage of apoptosis/necrosis were compared to the control. Error bars represent the standard error of the mean. *: p < 0.05; **p < 0.01; ***: p < 0.001; ****: p < 0.0001

Flow cytometry was performed to assess apoptosis and necrosis. Cells were seeded into 6-well plates, treated with 0, 4, 8 or 16 µM zerumbone and incubated for 48 or 72 h. All three cell lines showed a dose-dependent increase of apoptosis and necrosis (Fig. 2). After 24 h of incubation, apoptosis was significantly increased in SCC25 at 16 µM, and in Cal27 at 8 and 16 µM. While the amount of necrosis was not affected in SCC25 and FaDu after 24 h, it was significantly increased in Cal27 at 16 µM. After 72 h, apoptosis was significantly increased in SCC25 at 8 and 16 µM and FaDu at 16 µM. Furthermore, after 72 h necrosis was increased in all three cell lines at 16 µM and additionally in Cal27 at 8 µM. However, it should be noted that in this assay late apoptosis is not distinguishable from necrosis.

### Wound healing assay

**Fig. 3:**
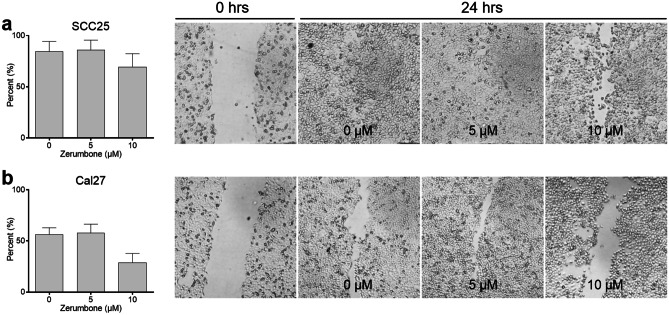
Wound healing assay of (**a**) SCC25 and (**b**) Cal27. Cells were photographed immediately after performing the scratch (0 h) and after 24 h of incubation with 0, 5, 10 µM zerumbone. Percentage of migration after 24 h of incubation was assessed. Error bars represent the standard error of the mean

Cell migration was investigated using the wound healing assay. Cells were seeded onto 6-well plates and a scratch was made prior to incubation with zerumbone at 0, 5, 10 and 20 µM for 24 h. There was a notable difference in the wound healing capacity in SCC25 and in Cal27 at 10—20 µM. In Cal27 the wound healing area has closed by 53% in the control group and 31% at 10 µM zerumbone. In SCC25 the wound healing area has closed by 84% in the control group and 69% at 10 µM zerumbone (Fig. 3). Although there was a significant reduction of wound healing in SCC25 and Cal27 at 20 µM, the overall cell density has markedly decreased due to cytotoxicity (data not shown). Results of FaDu were not reproducible due to tissue tearing when performing the scratch.

### Effect of zerumbone and cisplatin on cell viability

To determine a possible synergism with the chemotherapeutic agent cisplatin, cells were treated with cisplatin and zerumbone simultaneously at a ratio of 3:1. In Cal27 the combination with zerumbone led to an enhanced effect of cisplatin treatment. In SCC25 and FaDu zerumbone decreased the effect of cisplatin treatment. Next, the results were analyzed with the Combination Index (CI). CI < 1 indicates synergy, CI > 1 an antagonistic effect and CI = 1 represents an additive effect. Treatment combination in SCC25 and FaDu showed an antagonistic effect for all doses. The CI for Cal27 revealed a synergistic to additive effect up to the combination of 4.88 µM zerumbone and 1.63 µM cisplatin (CI 0.65—1.08) and an antagonism for higher doses (Fig. 1).

### Effect of zerumbone and radiation on cell viability and colony formation

**Fig. 4:**
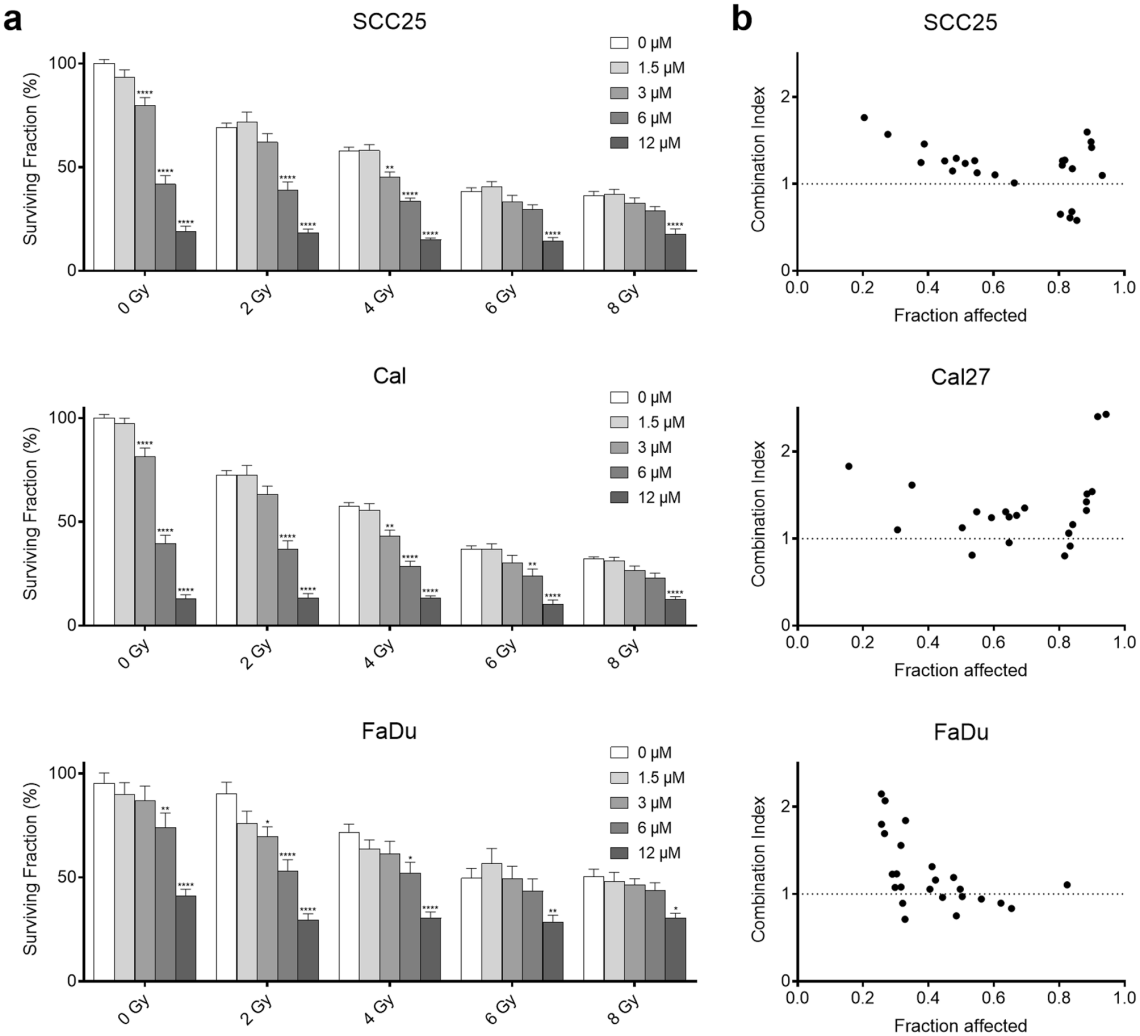
Combination of zerumbone and irradiation. (**a**) Survival (%) after treatment with zerumbone and radiation for 72 h. (**b**) Experimental CI values of zerumbone and radiation. CI < 1 indicates synergy, CI > 1 antagonism and CI = 1 represents an additive effect. Error bars represent the standard error of the mean. Significance levels were compared to the control group within the same radiation dose. *: p < 0.05; **p < 0.01; ***: p < 0.001; ****: p < 0.0001

To assess the short-term effect, cells were treated with zerumbone (0—96 µM) and irradiation (0—8 Gy) for 72 h. Treatment with zerumbone enhanced the radiation effect in all three cell lines at different concentrations (Fig. 4). When analyzed with the CI a synergistic effect was found in SCC25 for 12 µM zerumbone and 2—8 Gy (CI 0.57—0.68). Cal27 showed a slight to moderate synergism when 6—12 µM zerumbone were combined with 2—4 Gy (CI 0.80—0.95). FaDu showed a nearly additive or synergistic effect at 1.5—12 µM and 2—6 Gy (CI 0.71—1.1).

**Fig. 5:**
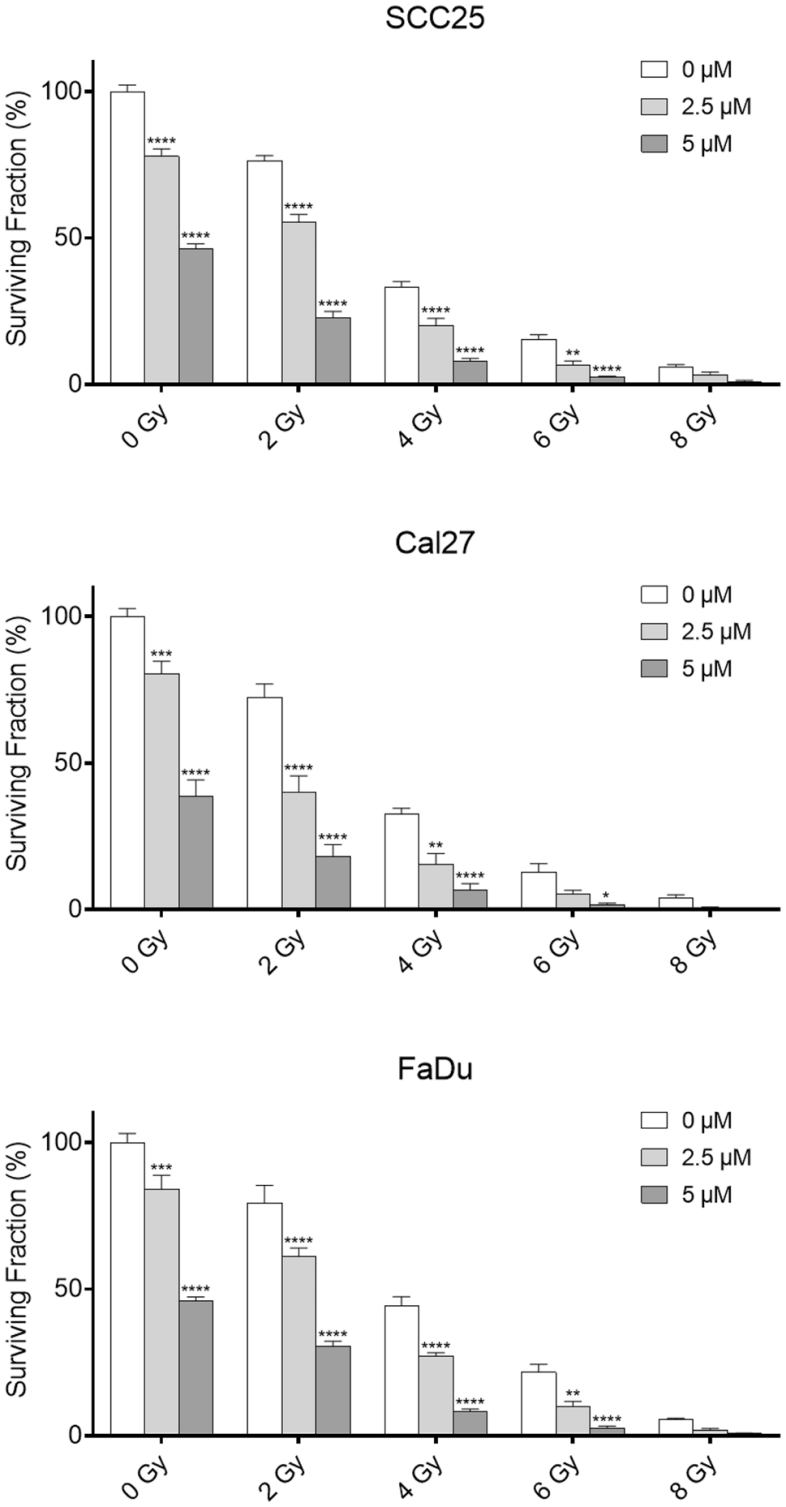
Clonogenic assay of SCC25, Cal27, and FaDu cells. Cells were treated with 0, 2.5 or 5 µM zerumbone and subsequently irradiated with 0, 2, 4, 6, or 8 Gy. All cell lines showed a significant inhibition of colony formation when analyzed with the linear-quadratic model by Franken et al. (all p < 0.001). Error bars represent the standard error of the mean. The indicated significance levels were additionally analyzed using a two-way ANOVA. Treated cells were compared to the control group within the same radiation dose. *: p < 0.05; **p < 0.01; ***: p < 0.001; ****: p < 0.0001

The long-term effect of combination treatment was assessed with the clonogenic assay. Treatment with zerumbone and/or radiation led to a decrease of clonogenic survival in all cell lines. Moreover, all three cell lines showed a significantly increased inhibition of colony formation at 2.5 and 5 µM (all p < 0.001, Fig. 5) when analyzed with linear regression according to Franken et al. [13].

## Discussion

HNSCC is one of the sixth most common carcinoma worldwide and survival rate only marginally improved over the last decades. Thus, new treatment options are needed to improve overall survival. The investigation of natural products has led to the discovery of several well-known chemotherapeutic agents. The ginger plant *Zingiber zerumbet* is commonly used in traditional medicine in Asia to treat a wide variety of symptoms and diseases. Zerumbone is the major component of its essential oil and has been researched in many studies in vivo and in vitro. It shows a cytotoxic effect in cancer cell lines such as lung cancer [15], colorectal cancer [16], leukemia [11] and oral cancer [9]. However, it has minimal to no effect on normal cells [9, 16, 17].

In this study, the effect of zerumbone on the HNSCC cell lines SCC25, Cal27 and FaDu was investigated. Zerumbone showed antiproliferative effects in all three cell lines with IC_50_ values between 4.4—9.2 µM. Additional flow cytometry analysis showed upregulation of apoptosis in all three cell lines. Migration was reduced in Cal27 at 10 µM. In concordance, Zainal et al. showed an antiproliferative effect in oral cancer cells between 0.8—4.9 µM and a proapoptotic and antimigratory effect [9].

Next, the combinatory effect of zerumbone and cisplatin at a ratio of 3:1 was investigated, since cisplatin is the standard chemotherapeutic agent in HNSCC treatment [18]. Combined treatment showed an antagonistic effect in SCC25 and FaDu. In Cal27 an additive to synergistic effect for low drug doses was found. However, for cancer therapy synergism at high concentrations is more useful in order to make the most of both substances [14]. Hence, we assume that zerumbone is no suitable candidate for combination with cisplatin in HNSCC. So far, only Hu et al. investigated the combinatory effect of zerumbone and cisplatin in vitro on lung cancer cells. In contrast to our study they demonstrated a significantly enhanced growth suppression when both substances were combined [15].

Another pillar of HNSCC treatment is radiotherapy. In this study, combination experiments of zerumbone and radiation showed an additive or synergistic effect at different concentrations in all three cell lines. Furthermore, the clonogenic assay showed a significantly reduced colony formation by combination of both treatments. Likewise, a radiosensitizing effect was found in colorectal carcinoma [16], prostate cancer [19] and glioblastoma cells [20]. Underlying mechanisms include inhibition of radiation-induced activation DNA double-strand break repair via inhibition of ataxia-telangiectasia mutated (ATM) and DNA-dependent protein kinase, catalytic subunit (DNA-PKcs) [16, 19]. Furthermore, treatment with zerumbone alone and in combination with radiation induced cell cycle arrest in G2/M, the most vulnerable phase for radiation and depletion of the cellular antioxidant glutathione [16].

In conclusion, zerumbone shows a cytotoxic and proapoptotic effect, and inhibits cell migration in HNSCC cell lines. Moreover, this is the first study to describe a radiosensitizing effect of zerumbone in HNSCC cell lines. These results implicate that zerumbone might be an attractive treatment option in HNSCC especially in combination with radiation. However, further preclinical and clinical studies are required to assess the full potential of zerumbone.

## Data Availability

The datasets generated and analyzed during the current study are available from the corresponding author on reasonable request.

## Abbreviations

CCK-8: Cell Counting Kit-8
CIS: Cisplatin
DMSO: Dimethyl sulfoxide
HNSCC: Head and neck squamous cell carcinoma
SEM: Standard error of the mean
ZER: Zerumbone
